# Husbands’ participation in birth preparedness and complication readiness and associated factors in Wolaita Sodo town, Southern Ethiopia

**DOI:** 10.4102/phcfm.v10i1.1471

**Published:** 2018-04-11

**Authors:** Minyahil Tadesse, Andualem T. Boltena, Benedict O. Asamoah

**Affiliations:** 1 School of Public Health, College of Health Sciences, Wolaita Sodo University, Ethiopia; 2 School of Public Health, Lund University, Sweden; 3 Social Medicine and Global Health, Department of Clinical Sciences, Lund University, Sweden

## Abstract

**Background:**

The poor emphasis on the role of husbands in birth preparedness and complication readiness (BPCR) is a major factor that should be addressed in tackling maternal mortality.

**Aim:**

To assess the level of husbands’ participation in BPCR and associated factors.

**Setting:**

Wolaita Sodo town, Southern Ethiopia.

**Methods:**

A community based cross-sectional study was conducted among 608 husbands of pregnant women and nursing mothers. Multivariate logistic regression model was used for the analysis.

**Results:**

Forty-five per cent of husbands studied had poor participation in BPCR. Out of the total husbands studied, 40% (235) did not identify transportation, 49% (291) did not accompany their wives to antenatal care (ANC) clinic, 59% (350) did not identify skilled birth attendant, 26% (155) did not identify health facility for delivery and 30% (179) did not save money for emergency. Only 42% (250) of husbands had awareness of emergency conditions, while 75% (444) did not make postpartum plan. Husbands who knew the place of birth of the baby [adjusted odds ratio (AOR) = 7.23; 95% confidence interval (CI): 2.98–17.54] and those who discussed with their wives about birth preparedness (AOR = 2.03; 95% CI: 1.37–3.02) were significantly more likely to participate in BPCR compared to those who did not.

**Conclusion:**

Participation of husbands in BPCR was poor in the study area. The level of participation in relation to selection of service provider and health facility, financial and transportation planning for delivery and identifying blood donor needs attention to achieve better husband participation in BPCR.

## Introduction

Maternal mortality is one of the most sensitive indicators of health disparity among low-, middle- and high-income countries and remains largely a major contributor to unmet public health concerns globally. Remarkable disparities in maternal deaths exist between and within countries, with wide variations between the rich and the poor, and urban and rural areas.^1,2^ For instance, in 2015, the risk of losing one’s life through pregnancy in the sub-Saharan African region was 1:36 compared to the global average lifetime risk of 1:180. At the country level, in 2015, lifetime risks of maternal deaths ranged from 1 death per 23 700 women in Greece to 1 death per 17 in Sierra Leone.^3^ The Fifth Millennium Development Goal (MDG 5), which sought a 75% reduction in every country’s maternal mortality ratio by 2015, lagged the farthest behind in developing countries.

Estimates from 2015 indicated that 546 maternal deaths per 100 000 live births were recorded in sub-Saharan Africa compared to 216 maternal deaths per 100 000 live births worldwide.^1,3^ With the new global sustainability agenda, more effort and marked acceleration in progress are needed to achieve the sustainable development goal (SDG) 3.1, which seeks to reduce the global maternal mortality ratio to less than 70 per 100 000 live births.^1^ The major causes of maternal deaths are severe bleeding, pregnancy-induced hypertension, infections, obstructed labour, unsafe abortion and other pre-existing conditions exacerbated by pregnancy. Generally, high fertility rate in sub-Saharan African countries coupled with economic, sociocultural, health systems and policy factors accounts for the high maternal mortality rate experienced in this region.^1,4^ Thus, most maternal deaths are preventable through access to and utilisation of effective modern contraception, good quality antenatal care (ANC), skilled birth attendants and postpartum care.^1,4^ However, even within countries in sub-Saharan Africa, there exist huge differences in maternal mortality between women from different socio-economic backgrounds.^2^ The huge burden of maternal mortality and morbidity and the health systems challenges in this region call for community involvement and strong family or partner support along the continuum of maternal care pathway.^5^ One such essential approach is birth preparedness and complication readiness (BPCR), a strategy that has the potential to promote active utilisation of skilled birth attendants and emergency obstetric care.^6^ Utilisation of skilled birth attendants and access to emergency obstetric care are proven interventions globally for reducing maternal mortality and saving the life of the newborns.

A number of strategies used a framework based on the primary healthcare principles where primary prevention (preventing a condition from occurring through education and services) was distinguished from secondary (detection and treatment of conditions early) and tertiary (treatment of conditions to reduce case fatality) preventions.^7^

Although husbands have an important role to play in BPCR, the systematic review by Mittenburg and colleagues in 2015 found that previous studies and interventions on BPCR focused mainly on the mothers. Although a few interventions have targeted husbands and mothers-in-law, most studies exclusively evaluated women’s behaviour as the primary outcome.^8^ The role of husbands in BPCR should include knowledge of key danger signs during pregnancy (e.g. severe vaginal bleeding at all stages), labour and childbirth (e.g. prolonged labour, convulsions and retained placenta), and during the postpartum period (e.g. foul-smelling vaginal discharge and high fever). Other roles include identifying transportation and health facility, arrangement for a skilled birth attendant, saving money for delivery and emergency, arrangement for blood donor, accompanying wife and making postpartum readiness plan for both the mother and the baby (which includes provision of food, drinks and the like to assist the mother in exclusive breastfeeding).^9^ A study on BPCR among recent fathers in Tanzania found that almost half of the husbands had saved money for delivery and emergency, whereas a very small proportion had identified transport and skilled birth attendant prior to delivery. In another study conducted in Uganda, it was found that factors such as formal education above secondary level, formal occupation and presence of complications were associated with male involvement in BPCR.^10^

Attention has long been drawn to the absence of men from previous reproductive health initiatives, although men play a major role in influencing women’s reproductive health, both positively and negatively.^11,12^ In order to address this daunting challenge, the 1994 International Conference on Population and Development (ICPD) in Cairo, Egypt, strongly recommended that special efforts should be made to emphasise men’s shared responsibility and promote their active participation in maternal health.^13^ Despite this fact, pregnancy and childbirth is regarded as exclusively women’s affair in most African countries.^14,15^ A male partner is rarely seen at ANC. A review by Ditekemena in 2012 found that health service factors such as harsh, critical behaviour and language used by health providers, financial constraints, lack of space to accommodate male partners at ANC clinics, long waiting time during ANC services and opening hours for ANC clinics are associated with poor male partner participation in ANC attendance. In addition, socio-demographic factors (such as age, education and profession) and sociocultural factors (such as negative perceptions about men attending ANC clinics and poor communication between men and their female partners) also account for poor male involvement.^16^ Nonetheless, men have social and economic power, and have more control over their partners. They decide the timing and conditions of sexual relations, family size and whether or not their spouses will utilise available health care services.^17,18^

Hence, involvement of male partner becomes even more critical in patriarchal societies if considerable improvement in maternal health outcome is to be realised. Strategies for involving men in maternal health services ought to aim at raising their awareness about emergency obstetric conditions and engaging them in BPCR.^19^ Male involvement will enable men to support their spouses to utilise emergency obstetric services early and the couple would adequately prepare for birth and make themselves ready for complications. Similarly, preparing for birth and being ready for complications would reduce all three phases of delay related to the continuum of maternal health care – delays in deciding to seek care, delays in reaching care and delays in receiving care – and thereby positively impact birth outcomes.^7,20,21^

Ethiopia continues to have one of the highest maternal mortality rates in the world, with an estimated maternal mortality of 353 per 100 000 live births in 2015.^22^ The health care delivery system in Ethiopia has three tiers, which are characterised by a *woreda* health setup that encompasses firstly the health centre and health post, secondly the district hospital and lastly the referral hospital. The number of ANC visits that the Government of Ethiopia recommends is the same as that of the World Health Organisation (WHO) recommendation, which is at least four. There is no cost for the ANC services provided for pregnant women.^23^ The role of men in maternal health care is seldom understood in Ethiopia. A study conducted in Tigray, Northern Ethiopia, revealed that 20% of husbands refused to use family planning methods.^24^ Another study from Tigray reported that only 21% of pregnant mothers were accompanied by their husbands to antenatal clinic, and 16% of husbands were found to disapprove ANC attendance.^25^ Because majority of the studies conducted in Ethiopia on BPCR were conducted among mothers,^26,27,28^ poor emphasis was given for the role of husbands. Therefore, the aim of this descriptive study is to assess husbands’ participation in BPCR in Wolaita Sodo town, Southern Ethiopia. We hope that this study will shed more light on the magnitude of husbands’ participation in an urban setting in Ethiopia.

## Research methods and design

### Study area and setting

A community-based cross-sectional study was conducted in Wolaita Sodo town, located in Southern Nations, Nationalities, and Peoples’ Region (SNNPR), which is 327 km from Addis Ababa, the capital of Ethiopia. It has three sub-city administrations consisting of 11 districts. The population of the town was estimated to be 100 755 in 2015, and it has one teaching referral hospital, a non-governmental hospital, seven private medium clinics and three governmental health centres.^28^ Antenatal care, delivery care and caesarean section services are offered free of charge by public health facilities to all women in the study area. According to the zonal health department report of Wolaita zone, the prevalence of health facility delivery in 2016 was 25.5%,^29^ which is lower than the national facility delivery rate of 26%.^30^

### Sample size and sampling procedure

The sample size was determined by using a formula for estimation of single population proportion with the assumption of 95% confidence level, an error margin of 5% and the prevalence of husbands’ participation in BPCR (60%) – taken from a previous study in a rural area.^2^ After considering a 10% non-response rate and multiplying by the design effect of 1.5, the total sample size was estimated to be 607. Multistage sampling technique was used. There were three sub-cities consisting of 11 districts in Wolaita Sodo town. Eight districts were randomly selected for this study. For each district, probability proportional allocation to sample size was made according to the total number of households with husbands of pregnant women and nursing mothers. Sampling frame of households with husbands of pregnant women and nursing mothers was known based on the ANC registration in the Wolaita Sodo town health office. Finally, systematic random sampling method was used to select 607 households with husbands of pregnant women and nursing mothers.

### Data collection

Data were collected by a pretested structured questionnaire, adopted from JHPIEGO,^8^ which was already prepared in English and then translated to Amharic.

Eight data collectors, based on their previous experience, were recruited and trained for data collection, and two trained public health officers supervised collection of the data. The research assistants read out the questions loudly and the husbands answered every question accordingly. If prospective participants (husbands of pregnant women or nursing mothers from identified households) were not found at home during the first visit, the data collectors made a second visit the next day to administer the questionnaire. Prior to data collection, all of the study subjects were oriented and well informed about the purpose of the study and their right to accept or refuse to participate in the interview.

### Data quality management and entry

Before the actual data collection, the questionnaire was pretested on a similar setting outside the study area. The data collectors and supervisors were trained for 2 days on principles, ethical considerations, procedures and details of the questionnaire. The principal investigator closely monitored the data collection process. Completed questionnaires were checked for their consistency and completeness every day and then entered into EPi-Info version 3.5.4 statistical software, and finally the data were exported to another statistical software package SPSS, version 20.0, for further cleaning and analysis. Statistical significance was set at *p* < 0.050 and 95% confidence interval (CI).

### Operational definition

#### Outcome variable

Husbands’ participation in BPCR was measured by nine items. For each item, those who responded ‘Yes’ scored 1 and those who responded ‘No’ scored 0. Then the mean score was computed as being four out of the nine items.

Good participation in BPCR: Those husbands who practised at least five elements of nine items (Table 2).

Poor participation in BPCR: Those husbands who practised at most four elements of nine items (Table 2). That means husbands who practised four or fewer elements of nine items were categorised as having poor participation in BPCR.

#### Socio-demographic variables

Husband’s age in years was categorised as ‘20–30 years’, ‘31–40 years’ and ‘41–50 years’.

Religion was categorised as ‘Protestant’, ‘Orthodox’, ‘Catholic’ and ‘Muslim’. Husband’s education was classified into four categories: ‘no formal education’, ‘primary education’, ‘secondary education’ and ‘college degree or diploma’. Husband’s occupation was categorised as ‘farmer’, ‘merchant’, ‘daily labourer’, ‘government employed’ and ‘other (taxi driver, farmer)’. Wife’s age in years was categorised as ‘< 20 years’, ‘20–29 years’ and ‘30–39 years’. Wife’s education was classified into four categories, similar to husband’s education. Wife’s occupation was classified as ‘housewife’, ‘merchant’, ‘daily labourer’, ‘government employed’ and ‘other (traditional spinning)’.

Family’s monthly income in Ethiopian birr (ETH birr): family income was classified into three categories (< 500, 500–1000, > 1000 ETH birr) using percentiles. The source of information on BPCR was indicated as radio, television, newspaper and discussions with wives, neighbours and health professionals.

### Ethical consideration

Ethical clearance was obtained from the Research and Ethical Committee of Wolaita Sodo University, School of Public Health. Informed verbal consent was obtained from each study subject prior to data collection, and the purpose of the study was explained to the respondents in advance. Confidentiality of the information and privacy of the respondents were maintained. During data collection, each of the study participants was communicated that their participation would be voluntary and that they can quit from the study at any time even after the interview has started.

## Results

A total of 592 subjects participated in the study, yielding a response rate of 98%. Forty-six per cent of husbands (273) were in the age range of 31–40 years, while slightly above half (52%, 310) were followers of Protestant Christianity. Only 37% (218) of husbands attained educational level of a college degree or diploma, while 28% (163) were government employed by occupation. Regarding socio-demographic characteristics of wives, 60% (355) were aged 20–29 years, 58% (345) were housewives by occupation and 30% (180) had attended primary education. About 64% (381) of the households had income more than 1000 Ethiopian birr (≈ 45 USD) per month. Regarding husbands’ sources of information about BPCR, about half of the husbands (51.4%, 304) had heard about it from radio, whereas 43.9% (260) had heard about it from television. Moreover, only 28.0% (166) of the husbands had read about BPCR from newspapers, while 45.9% (272) reported discussing BPCR with their wives. Nearly one-third of the husbands (31.6%) reported discussing with their neighbours, while nearly one-quarter (22.5%) reported discussing BPCR with a health professional (Table 1).

**TABLE 1:** Socio-demographic characteristics of (*n* = 607) husbands in Wolaita Sodo town, Southern Ethiopia, December 2014 to January 2015.

Variables	Result	Frequency (#)	%
Husband’s age in years	20–30	250	42.2
	31–40	273	46.1
	41–50	69	11.7
Religion	Protestant	310	52.4
	Orthodox	211	35.6
	Catholic	31	5.2
	Muslim	40	6.8
Husband’s education	No formal education	50	8.4
	Primary education	165	27.9
	Secondary education	159	26.9
	College degree or diploma	218	36.8
Husband’s occupation	Farmer	17	2.9
	Merchant	175	29.6
	Daily labourer	113	19.1
	Government employed	163	27.5
	Other†	124	20.9
Wife’s age in years	< 20	75	12.7
	20–29	355	60
	30–39	162	27.4
Wife’s education	No formal education	97	16.4
	Primary education	180	30.4
	Secondary education	148	25
	College degree or diploma	167	28.2
Wife’s occupation	House wife	345	58.3
	Merchant	78	13.2
	Daily labourer	24	4.1
	Government employed	102	17.2
	Other‡	43	7.2
Family’s monthly income in Ethiopian birr	< 500	88	14.9
	500–1000	123	20.8
	> 1000	381	64.4
Husband’s source of information on birth preparedness and complication readiness	Heard from radio	304	51.4
	Heard from television	260	43.9
	Read from newspaper	166	28.0
	Discussed with wives	272	45.9
	Discussed with neighbour	187	31.6
	Discussed with health professional	133	22.5

† , taxi driver, farmer.

‡ , traditional spinning.

Twenty-five per cent (146) of the husbands did not save money for delivery and postpartum and only one in five (108) husbands identified blood donor. Among the husbands studied, 45% (268) had poor participation in BPCR during pregnancy, delivery and postpartum period. Out of the total husbands studied, 40% (235) did not identify transportation, 49% (291) did not accompany their wives to ANC clinic, 59% (350) did not identify skilled birth attendants, 26% (155) did not identify health facility for delivery and 30.2% (179) did not save money for emergency, and only 42% (250) had awareness of emergency condition, while 75% (444) did not make postpartum plan (Table 2).

**TABLE 2:** Husbands’ participation in birth preparedness and complication readiness in Wolaita Sodo town, Southern Ethiopia, December 2014 to January 2015.

Variables	Result	Frequency (*n*)	%
Identified transportation	Yes	357	(60.3)
	No	235	(39.7)
Saved money for delivery†	Yes	446	(75.3)
	No	146	(24.7)
Accompanied wife to antenatal care	Yes	301	(50.8)
	No	291	(49.2)
Identified blood donor	Yes	108	(18.2)
	No	484	(81.8)
Identified skilled birth attendant	Yes	242	(40.9)
	No	350	(59.1)
Identified health facility	Yes	437	(73.8)
	No	155	(26.2)
Saved money for emergency‡	Yes	413	(69.8)
	No	179	(30.2)
Aware of emergency conditions	Yes	250	(42.2)
	No	342	(57.8)
Made postpartum plan	Yes	148	(25.0)
	No	444	(75.0)
Husband’s participation in birth preparedness and complication readiness	Good participation (≥ 5 items)	324	(54.7)
	Poor participation (< 5 items)	268	(45.3)

† , known costs usually associated with delivery, such as transport to health facility.

‡ , extra money for other unforeseen occurrences, which may occur along the continuum of the pregnancy.

Socio-demographic characteristics such as husband’s education (college degree or diploma) and wife’s age (20–29 years) and occupation (being government employed) were found to be statistically significantly associated with increased husbands’ participation in BPCR in the bivariate analysis. Among these variables, only having wives aged 20–29 years was (adjusted odds ratio [AOR] = 2.68; 95% CI: 1.40–5.12) found to be significantly associated with increased husbands’ participation in BPCR in the multivariate analysis. Regarding husbands’ participation, those who accompanied wives for ANC visits (AOR [for husbands who followed their wives’ ANC visits four times] = 7.12; 95% CI: 3.74–13.58), those who became aware of their wives’ ANC visit in the second trimesters (AOR = 2.54; 95% CI: 1.01–6.39) and those who planned the place of birth of the baby (AOR = 7.23; 95% CI: 2.98–17.54) were significantly more likely to participate in BPCR.

Sources of information and husbands’ participation in BPCR were found to be significantly associated in the bivariate analysis, but only husbands who discussed with their wives about BPCR showed an association in multivariate analysis (AOR = 2.03; 95% CI: 1.37–3.02) (Table 3).

**TABLE 3:** Factors associated with husbands’ participation in birth preparedness and complication readiness in Wolaita Sodo town, Southern Ethiopia, December 2014 to January 2015.

Variables	Result	Participation in BPCR		COR (95% CI)	AOR (95% CI)
		Poor participation	Good participation		
Husband’s education	No formal education	27	23	1	-
	Primary education	98	67	0.80 (0.42–1.51)	0.73 (0.32–1.70)
	Secondary education	69	90	1.53 (0.80–2.89)	1.00 (0.43–2.32)
	College degree or diploma	74	144	2.28 (1.22–4.25)†	1.24 (0.52–2.93)
Wife’s age in years	< 20	43	32	1	-
	20–29	158	197	1.67 (1.01–0.77)†	2.68 (1.40–5.12)‡
	30–39	67	95	1.90 (1.09–3.31)	1.60 (0.91–2.82)
Wife’s occupation	House wife	161	184	1	-
	Merchant	36	42	1.02 (0.62–1.67)	0.69 (0.38–1.25)
	Daily labourer	16	8	0.43 (0.18–1.04)	0.46 (0.16–1.33)
	Government employed	36	66	1.60 (1.01–0.53)†	0.90 (0.48–1.65)
	Other	19	24	1.10 (0.58–2.09)	0.63 (0.28–1.39)
Husband who followed the number of times his wife made ANC visit§	One time	53	54	4.12 (2.27–7.45)	3.91 (2.02–7.59)
	Two times	41	106	10.45 (5.84–8.70)	8.99 (4.70–17.21)
	Three times	36	57	6.40 (3.45–11.88)	5.73 (2.91–11.31)
	Four times	45	84	7.54 (4.21–3.51)†	7.12 (3.74–13.58)‡
	I don’t know	93	23	1	-
Husband being aware of the trimesters when his wife made first ANC visit	1–3	92	136	6.50 (3.74–11.30)	1.86 (0.73–4.74)
	4–6	82	152	8.15 (4.68–14.20)	2.54 (1.01–6.39)‡
	7–9	6	16	11.73 (4.08–3.74)†	2.60 (0.63–10.61)
	I don’t know	88	20	1	-
Husband who knows the planned place of birth of the baby	Health facility	218	317	10.38 (4.62–3.33)†	7.23 (2.98–17.54)‡
	Home	50	7	1	-
Husband who heard from radio about BPCR	Yes	133	191	1.97 (1.41–2.73)†	1.14 (0.73–1.76)
	No	155	113	1	-
Husband who heard from television about BPCR	Yes	77	183	3.21 (2.28–4.54)†	1.76 (1.16–2.66)
	No	191	141	1	-
Husband who read from newspaper about BPCR	Yes	46	120	2.83 (1.92–4.19)†	1.80 (1.12–2.89)
	No	222	204	1	-
Husband who discussed with his wife about BPCR	Yes	81	191	3.31 (2.35–4.66)†	2.03 (1.37–3.02)‡
	No	187	133	1	-
Husband who discussed with neighbour about BPCR	Yes	23	58	2.32 (1.39–3.88)†	1.04 (0.55–1.98)
	No	245	266	1	-
Husband who discussed with health professional about BPCR	Yes	29	84	2.88 (1.82–4.56)†	1.30 (0.70–2.38)
	No	239	240	1	-

BPCR, in birth preparedness and complication readiness; CI, confidence interval; ANC, antenatal care; COR, crude odds ratio; AOR, adjusted odds ratio.

Statistical significance was set at *p*< 0.050.

† , factors associated with husbands’ participation in birth preparedness and complication readiness.

‡ , independent predictors of husbands’ participation in birth preparedness and complication readiness.

§ , it is the number of times the husband went to health facility with his wife to follow her ANC visit.

## Discussion

This study has shown that approximately half of the husbands were either not participating at all or had poor participation in BPCR as they were practising four or less elements of the nine items during pregnancy, delivery and postpartum period. This finding indicates higher percentage of husbands not participating in BPCR compared with the study conducted in Mekele town, Northern Ethiopia, in which the husbands who participated in BPCR constituted 40%.^31^ In this study, one in four husbands did not save money for delivery and postpartum. Having no money during delivery and postpartum could mean that the mother is subject to high risk of birth complications and death from unskilled birth attendants. The fact that only one in five husbands identified blood donor can be seen as a threat to maternal survival as bleeding during delivery and post-delivery is the topmost cause of maternal mortality. A study conducted in Uganda found that one in 10 women had blood loss during delivery. This could drastically increase the risk of mortality^32^ as haemorrhage remains one of the topmost causes of maternal mortality,^1^ and most deaths occur within 24–48 h of delivery because of prolonged blood loss.^33^ On the other hand, the donated blood should be carefully managed and screened for blood-borne infections such as HIV and hepatitis.

Every two out of five husbands did not identify transportation, which implies that during emergency it becomes problematic to transport the mother in time to avoid birth complications or even death. Half of the husbands did not accompany their wives, making them visit the ANC clinic unaccompanied. This is higher compared to the study conducted in Northern Ethiopia.^31^ A quarter of the husbands failed to identify the health facility where their wives would deliver and three in five husbands did not identify a skilled birth attendant in this study. This is very worrisome because giving birth with the assistance of a skilled birth attendant is a well-known intervention that drastically reduces maternal mortality and related morbidities.^34,35^

About a quarter of the husbands did not save money for emergency, which could indicate not only poor financial planning but also inadequate financial resources to enable saving for such emergencies. Lack of financial resources in emergency situations coupled with lack of other BPCR factors such as having made no pre-arrangements for transport, not being aware of emergencies and having no postpartum plan could form a cluster of risk factors that could eventually lead to the death of the mother because of prolongation of the first two delays in the continuum of care: delay in deciding to seek care and delay in reaching health care facility. Hence, saving money for transportation and essential expenses for delivery and postpartum as well as for unforeseen occurrences as early as possible can save the woman’s life. Thus, interventions that support husbands to make such preparations could also help to save the life of the mother.

This study also examined factors associated with husbands’ participation in BPCR. Husbands with wives younger than 20 years were less likely to participate in BPCR as compared to husbands with wives aged 20–29 years and 30–39 years. This finding is consistent with a study conducted in Kinshasa.^36^ This could be attributed to the lack of previous experience of complications that wives faced during delivery or postpartum period as for most of the mothers younger than 20 years it could probably be their first birth experience. Another reason could be the lack of active participation in ANC by husbands of these wives. Although wives are counselled by health professionals during ANC to bring their husbands, for most teenage couples, sociocultural factors such as stigma and economic factors could deter husbands from actively participating in ANC attendance. Husbands who knew the place of birth of the baby and reported it to be a health facility were about seven times more likely to actively participate in BPCR compared to their counterparts whose wives gave birth at home. This finding is consistent with a study conducted in Northern Uganda.^37^ Husbands whose wives gave birth at health facilities were just below 40%. This is well below the average number of births attended by skilled health professionals in the African region.^1^ Having such low level of husbands who have their wives delivering at a health facility implies that the majority of pregnant women are at high risk of maternal mortality because of lack of skilled birth attendants.

Association between sources of information and husbands’ participation in BPCR was investigated and all the variables showed an association in the bivariate analysis. However, in the multivariate analysis, only husbands who discussed with their wives about BPCR were found to be significantly more likely to participate (Table 3). This could be because of lack of provision of health information to husbands regarding the utilisation of maternity care and also a lack of open discussion between husbands and wives related to their reduced BPCR. Moreover, usually husbands are the major decision makers in most household issues. Thus, discussion with the husbands regarding the need to be prepared for the birth and subsequent emergencies could have a positive influence on the women’s maternity care services. Unfortunately, in this study, slightly less than half of the husbands did not discuss BPCR with their wives.

### Strength and limitations of the study

This study presents a unique perspective to BPCR by evaluating it from the husbands’ perspective. The use of standard tool, pretesting of the questionnaire before actual data collection and training and supervision to control quality of the data add to the strength of this study. Nearly all the subjects were involved in the study.

This study utilised a cross-sectional design, which may present difficulties in ascertaining the direction of causality between the variables analysed. Therefore, caution needs to be taken in the interpretation of the findings with regard to causality. The study might be vulnerable to reporting bias, response bias and selection bias. Social desirability bias could arise from the fact that most men in this context would want to uphold or affirm social norms and masculinity values regarding the role of men as providers for the family and are potentially more likely to overreport their preparedness and readiness prior to delivery. However, we do not think that this would be a big problem in our study because we used a standardised questionnaire with multiple questions to assess BPCR. The lack of adjustment for unknown potential confounders in the multivariate regression analysis is also a limitation. This study was conducted in a town (urban setting), which limits its generalisability to rural and similar settings.

## Conclusion

Participation of husbands in BPCR was poor in the study area. In relation to the level of preparation and participation, saving money for delivery and identifying blood donor were the lowest. Therefore, interventions should be targeted towards improving male participation through promoting targeted policies and advocacies across all relevant stakeholders’ levels, including community education for men and women. Efforts should be made by the health care system to assist and welcome both men and women at ANC and delivery care.
